# Tumor-expressing PD-L1 regulates NT5E expression through MAPK/ERK pathway in triple-negative breast cancer

**DOI:** 10.32604/or.2025.061637

**Published:** 2025-06-26

**Authors:** CHENG CHENG, CHAO SHI, SHANG WU, WEIXING WU, JINGPING LI, SINUO GAO, MENG HAN, YIMIN WANG, XIANGMEI ZHANG, YUNJIANG LIU

**Affiliations:** 1 Breast Center, The Fourth Hospital of Hebei Medical University, Shijiazhuang, 050000, China; 2 Hebei Provincial Key Laboratory of Tumor Microenvironment and Drug Resistance, Hebei Medical University, Shijiazhuang, 050000, China; 3 General Surgery, The First Hospital of Qinhuangdao, Qinhuangdao, 066000, China; 4 Hebei Provincial Cancer Institute, The Fourth Hospital of Hebei Medical University, Shijiazhuang, 050000, China

**Keywords:** Breast cancer, Ecto-5′-nucleotidase (NT5E), Programmed cell death ligand 1 (PD-L1), ERK, Metastasis, Proliferation

## Abstract

**Objectives:**

While programmed cell death 1 (PD-1) inhibitors have improved cancer treatment, the function and mechanisms of programmed cell death ligand 1 (PD-L1), particularly when expressed by cancer cells, remain unclear. This study aims to explore the role of PD-L1 within breast cancer cells and identify key targets for future immunotherapy.

**Methods:**

RNA-seq was performed on breast cancer cells with silenced PD-L1 to screen for differentially expressed genes, followed by bioinformatics analysis. Clinical specimens from breast cancer patients undergoing primary surgery without preoperative treatment were collected, along with *in vitro* analysis to validate the potential mechanism.

**Results:**

RNA-seq data revealed a significant positive correlation between Ecto-5′-nucleotidase (NT5E) expression and PD-L1. Bioinformatics analysis corroborated this positive correlation. Immunohistochemistry staining demonstrated higher NT5E expression associated with increased lymph node metastasis. High expression of the NT5E gene was associated with poor overall survival (OS) in breast cancer patients, as determined by KM plotter analysis. Following PD-L1 gene silencing by siRNA in breast cancer cells, NT5E mRNA and protein expression significantly decreased. Conversely, no significant changes were observed in PD-L1 expression after NT5E gene silencing. *In vitro* experiments confirmed that cancer cell proliferation and metastasis abilities were significantly reduced by either PD-L1 or NT5E gene down-regulation. Western blotting demonstrated that PD-L1 expressed by cancer cells regulates NT5E expression through the MAPK/ERK signaling pathway.

**Conclusion:**

This study proposes a potential mechanism wherein tumor-expressing PD-L1 regulates NT5E through the MAPK/ERK pathway. Down-regulation of PD-L1 or NT5E can significantly inhibit the proliferation and metastatic ability of cancer cells, potentially providing practical therapeutic targets and prognostic markers for combined PD-L1 immunotherapy in breast cancer.

## Introduction

Breast cancer represents the most prevalent disease among women globally [1]. Triple-negative breast cancer (TNBC), a distinct subtype, is marked by the lack of estrogen receptors (ER), progesterone receptors (PR), and human epidermal growth factor receptor 2 (HER2). This subtype is associated with high invasiveness, significant metastatic potential, recurrence tendency, and poor prognosis [2]. Recently, immunotherapy has gained prominence as a vital treatment strategy for TNBC, focusing on obstructing the action of inhibitory immune checkpoint proteins and enhancing T-cell activation to foster anti-tumor immune reactions [3]. Programmed cell death protein 1 (PD-1, also referred to as PDCD1 and CD279) was first discovered by Ishida et al. in apoptotic tumors of mouse T cells [4]. PD-1 is a transmembrane protein widely present on the surface of activated immune cells, such as T cells, B cells, and monocytes. It negatively affects the immune response by binding to its ligands, programmed cell death ligand 1 (PD-L1) and programmed cell death ligand 2 (PD-L2). Nevertheless, when tumor cells exhibit PD-L1 or PD-L2, they can engage with PD-1 on T cells, thus hindering T cell activation and enabling immune evasion in cancer [5–7]. This mechanism has significantly contributed to the initiation and progression of malignancies.

Historically, the majority of research has focused on the molecular interactions and modifications between PD-1 and PD-L1 in the development of novel tumor immunotherapy approaches. However, recent investigations have revealed that PD-L1, beyond its role in facilitating immune evasion by tumor cells, is also recognized as a significant effector molecule. It has been shown to transduce intrinsic signals in tumor cells, promoting tumor progression independently of immune interactions [8–10]. Although PD-L1 is recognized for its role in regulating immune checkpoints, research investigating its function in tumor cells, especially in breast cancer, is still scarce. The precise mechanisms of PD-L1 action within breast cancer cells are yet to be fully elucidated.

The mechanisms of PD-L1 action within breast cancer cells remain unclear. While PD-1 inhibitors enhance cancer treatment efficacy, the function and mechanisms of PD-L1, particularly when expressed by cancer cells, are not fully understood [11,12]. Current literature highlights the necessity for novel immunotherapeutic approaches for TNBC. Ecto-5′-nucleotidase (NT5E, also known as CD73) [13] is implicated in modulating anti-tumour immune responses. NT5E is a 70 kDa glycosylated protein anchored to the extracellular surface of the plasma membrane by a glycosylphosphatidylinositol (GPI) anchor and is overexpressed in various tumors, including breast cancer. NT5E primarily catalyzes the conversion of extracellular 5′-AMP to adenosine (ADO) through adenosine receptors (A1, A2A, A2B, and A3AR), thereby regulating diverse physiological responses. Recent studies show that high glucose levels can enhance PD-L1 expression in human glioblastoma cells, affecting immune responses in the tumor microenvironment. This suggests a potential link between PD-L1 expression and tumor cell energy metabolism [14]. NT5E participates in the metabolism of adenosine, implying its potential influence on the tumor microenvironment and the function of immune cells. Consequently, NT5E emerges as a compelling candidate for investigating the functional dynamics of PD-L1. A relationship has been observed between NT5E and PD-L1 expression in various tumor types, including colon adenocarcinoma, lung adenocarcinoma, head and neck squamous carcinoma, and pancreatic adenocarcinoma [15]. The upregulation of NT5E correlates with aggressive cancer phenotypes, drug resistance, and pro-tumor activity. Recent findings have highlighted NT5E’s role in promoting the growth and metastasis of human breast cancer cells [16,17].

This investigation seeks to elucidate the function of tumor cell-expressing PD-L1 in breast cancer and its underlying mechanisms, with a particular emphasis on identifying NT5E as a crucial target. The findings are expected to yield significant insights into the regulatory mechanisms at the intracellular level and the resistance pathways related to tumor immunotherapy targeting PD-L1. The aim is to suggest creative methods for discovering new tumor biomarkers and formulating combination therapies, which will enhance early detection and enable personalized treatment strategies for patients.

## Materials and Methods

### Breast cancer patients and specimens

This study comprised 39 female breast cancer patients who underwent primary surgical treatment without preoperative therapy or distant metastases at the Breast Center of the Fourth Hospital of Hebei Medical University from January 2021 to May 2023 (Table A1). All patients received standardized adjuvant chemotherapy or hormone therapy postoperatively. The analysis excluded patients with bilateral breast cancer, occult breast cancer, major organ dysfunction incapable of receiving standard chemotherapy, and metastases at enrollment. The included patients had complete clinical-pathological data and follow-up information. All study participants provided signed consent forms after receiving detailed information about the research protocol, with voluntary participation confirmed before any study-related procedures were initiated.

### Cell culture, synchronization, and transfection

MDA-MB-231 and BT-549 human breast cancer cell lines were sourced from Shanghai Zhongqiao Xinzhou Biotechnology (Shanghai, China). MCF-7, SK-BR-3, MDA-MB-468, and T-47D cell lines were procured from Wuhan Pricella Biotechnology. All cell lines were verified to be Mycoplasma-free and authenticated using STR profile analysis one month before experiments. Cell cultures were maintained in high-glucose DMEM (Gibco, C11995500BT, Carlsbad, CA, USA) enriched with 10% heat-inactivated fetal bovine serum (Gibco, 10091-148) and 1% penicillin-streptomycin solution (Sigma-Aldrich, P0781, Saint Louis, MO, USA). The cells were incubated under standardized conditions at 37°C with 5% CO_2_ and 95% relative humidity. Selection of cells for target gene knockdown was based on the expression levels of specific target proteins across different cell types. For the transient transfection of siRNA, both the siRNA and the negative control (NC) were meticulously designed and procured from GenePharma (Shanghai, China). The most effective siRNA sequences were: siPD-L1: 5′-CUGAGAAUCAACACAACAATT-3′, siNT5E: 5′-GGAAUCGUUGGAUACACUUTT-3′ and NC: 5′-UUCUCCGAACGUGUCACGUTT-3′. For gene silencing experiments, cells in culture dishes incubated until reaching 50%–60% monolayer density. Transient transfection of siRNA duplexes was performed utilizing Lipofectamine 2000 transfection reagent (Invitrogen, 11668019, Carlsbad, CA, USA), following the supplier’s recommended protocol with optimized reagent-to-siRNA ratios.

### RNA extraction and quantitative real-time PCR analysis

Total RNA from cells isolation was performed utilizing TRIzol reagent (Invitrogen, 15596026, Carlsbad, CA, USA) and subsequently converted into cDNA with the TUREscript RT Master Mix reverse transcription kit (Aidlab, AT341, Beijing, China). cDNA amplification was carried out using the GO Taq qPCR Master Mix (Promega, A6002, Madison, WI, USA). The qRT-PCR primer sequences (Sangon Biotech, Shanghai, China) were as follows: Human PD-L1 (F: 5′-GACCACCACCACCAATTCCAAG-3′; R: 5′-TTAGTTGTTGTGTTGATTCTCAGTGTG-3′), human NT5E (F: 5′-CCCATTCTTCTAAACAGCAGCATTC-3′; R: 5′-TGATTGAGAGGAGCCATCCAGATAG-3′), and human GAPDH (F: 5′-GTGGACCTGACCTGCCGTCTAG-3′; R: 5′-GAGTGGGTGTCGCTGTTGAAGTC-3′). The thermal cycling protocol was established as follows: 95°C for 10 min, 40 cycles of: denaturation: 95°C for 15 s, primer annealing: 58°C for 30 s, extension: 72°C for 30 s, final elongation: 72°C for 10 min. Quantitative analysis of gene expression was performed through comparative CT quantification (2^−ΔΔCT^ method), with normalization to endogenous reference genes to ensure accurate relative quantification of target transcript levels across experimental conditions.

### RNA-seq analysis

Total cellular RNA was performed utilizing TRIzol reagent. Five pairs of TNBC MDA-MB-231 were prepared in knockout and negative control groups. RNA sequencing was conducted by Lianchuan Biotechnology. (LC-Bio Technology, Hangzhou, China). RNA from all samples was initially isolated and purified. The RNA integrity was assessed and validated based on the criteria. mRNA with PolyA (polyadenylate) was specifically captured through two rounds of purification. The isolated mRNA was fragmented and reverse-transcribed to cDNA. Subsequently, cDNA fragments were ligated to index adapters and amplified by PCR to generate libraries. The constructed library underwent to paired-end sequencing. Raw data were quality-controlled to eliminate low-quality reads and adaptor sequences. Subsequent bioinformatics investigations included comprehensive transcriptomic profiling, pathway enrichment evaluation through Gene Set Enrichment Analysis (GSEA), identification of differentially expressed genes (DEGs), and functional annotation analysis to elucidate biological processes and molecular pathways.

### Bioinformatics analysis

The raw data from transcriptome sequencing were processed. The criteria for identification of differentially expressed genes were |log_2_ Fold Change (FC)| ≥ 1 and *q* < 0.05, and visualized through a heatmap. Furthermore, a volcano plot was constructed with log_2_(FC) on the *X*-axis and −log_10_(*q*-value) on the *Y*-axis to illustrate top 20 differentially expressed genes facilitating the selection of potential downstream genes.

The TCGA database represents one of the most comprehensive and well-characterized cancer databases available. RNA sequencing data from the TCGA-breast cancer project were obtained and organized from the TCGA database. Data in Transcripts Per Million (TPM) format data and clinical information were extracted. The data underwent log_2_(value + 1) transformation, and results were visualized using the ggplot2 package in R (version 4.2.1). Correlation analysis, employing the Spearman statistical method, was conducted to identify downstream genes. Furthermore, survival analysis was performed using R packages survival (version 3.3.1), survminer (version 0.4.9), and ggplot2 (version 3.3.6) to execute Cox regression and generate Kaplan-Meier curves, assessing the impact of NT5E mRNA expression on overall survival (OS).

RNA-seq data along with clinical details for 615 breast cancer patients were sourced from the TCGA database. Normal adjacent tissue samples and sequencing data lacking clinical details were excluded from the analysis. (Table A2). Using the median NT5E expression value as a cutoff, the patient cohort was stratified into high- and low-expression subgroups to systematically evaluate potential correlations between NT5E transcriptional levels and clinicopathological details. The relationship between NT5E and PD-L1 was assessed through integration of RNA-seq data and bioinformatics analysis results. However, the database results only provide partial information regarding NT5E at the gene level and its correlation with PD-L1, and the database data may not fully represent the situation of breast cancer in our country. Therefore, we gathered 39 breast cancer samples from our hospital for the purpose of validation.

### Immunohistochemistry (IHC) assay

Immunohistochemical analysis was conducted on paraffin-embedded tumor tissues obtained from 39 breast cancer samples. Antigen retrieval was conducted by heating slides in EDTA (ethylenediaminetetraacetic acid) (pH 8.0). Prior to antibody incubation, treat the samples with 0.1%–0.5% Triton X-100 (Solarbio, T8200, Beijing, China) to enhance membrane permeability. Following the inactivation of endogenous peroxidases through incubation with 0.3% hydrogen peroxide solution, tissue sections were treated with a protein-blocking agent. Specimens were incubated for 14–16 h at 4°C with primary antibodies: PD-L1 antibody (1:100; Proteintech, 28076-1-AP, Wuhan, China) and NT5E antibody (1:100, Boster, A02120-3, Beijing, China). Immunodetection was then performed using biotinylated anti-rabbit (1:1000, Bioworld, Nanjing, China, BA1054) immunoglobulin for 30 min, followed by staining with 3,3′-diaminobenzidine chromogen (ZSBIO, ZLI9018, Beijing, China) as a substrate for visualization and Harris hematoxylin (BASO, BA4097, Zhuhai, China) for counterstaining. The slides were subsequently dehydrated and mounted with neutral gum. Positive and negative staining controls were carried out using paraffin-embedded tonsil sections, utilizing the same antibody in conjunction with a corresponding isotype-matched negative control antibody. IHC results were interpreted using a scoring system combining staining intensity and percentage of positive cells. Scores ≤4 were classified as negative, while scores of >4 were considered positive. Two experienced pathologists independently evaluated the IHC results to ensure accuracy and reliability.

### Cell proliferation and colony formation assays

4 × 10^3^ cells per well were seeded in 96-well plates and incubated for 6 h. Proliferation was evaluated using the Cell Counting Kit-8 (MedChemExpress, HY-K0301, Monmouth Junction, NJ, USA), measuring the OD at 450 nm. Proliferation was evaluated at 24, 48, 72, and 96 h. For assessment of clonogenic potential, a defined number of cells (2 × 10^3^ cells/well) were seeded into 6-well culture plates and maintained under standard growth conditions to allow colony development. Following a 14-day incubation period, cell colonies were immobilized using 4% paraformaldehyde solution for 20 min, followed by staining with 1% crystal violet solution (Solarbio, G1062, Beijing, China) for 10 min to visualize and quantify colony formation. The colonies were subsequently photographed and analyzed.

### Transwell assay

Transwell chambers (BD Biosciences, 3472, San Jose, CA, USA) were employed for the invasion assessment, whereas the migration assay was conducted without the use of Matrigel. For the invasion assay, Matrigel (BD 354234, Biosciences, USA) was uniformly applied to the culture chamber and incubated overnight at 37°C. 4 × 10^4^ cells are grown in per chamber and and incubated for 24 to 48 h. Following crystal violet staining, the cells were observed and photographed using an inverted phase contrast microscope (Leica, DFC295, Buffalo Grove, IL, USA). The number of cells was quantified, and the invasion and migration data were subsequently analyzed and graphically represented.

### Wound healing assay

Cells after gene silencing with siRNA were seeded at 2 × 10^5^ per chamber in 6-well plates. Following a 24-h incubation period, two vertical lines were drawn using a gun tip. The scratched area was imaged at 0 and 24 h post-injury using a microscope. The scratch healing rate (%) was determined using the formula [(0 h scratch area −24 h scratch area)/0 h scratch area] × 100%.

### Western blotting analysis

Cells were lysed with RIPA protein lysate (Solarbio, R0010, Beijing, China) (with selective addition of phosphatase inhibitors as required), incubated on ice for 30 min, centrifuged and supernatant was collected. Samples were subjected to analysis via 10% SDS-PAGE (Epizyme Biotech, PG212, Shanghai, China). Electrophoretically separated proteins were transferred to polyvinylidene fluoride (PVDF) membranes (MilliporeSigma, IPVH00010, Darmstadt, Germany). The membranes were blocked with blocking solution (Report, RW0501, Tianjin, China) for 1 h and incubated for 14–16 h at 4°C with antibodies against PD-L1 (1:1000; Proteintech, 28076-1-AP), NT5E (1:1000, Boster, A02120-3), MEK (1:500, Boster, BM4055), p-MEK (1:500, Boster, BM4690), ERK (1:500, Bioworld, AP0485), p-ERK (1:500, Bioworld, BS4759), and GAPDH (1:10000; Proteintech, 60004-1-Ig). Subsequently, each membrane was rinsed three times with Tris-buffered saline-Tween buffer (Epizyme biotech, TF103), with each wash lasting 10 min. The secondary antibodies used were: anti-mouse (1:1000, Bioworld, BA1050, China) and anti-rabbit (1:1000, Bioworld, BA1054), which were incubated with the membrane for 2 h. Following the washing procedure outlined for the primary antibody, specific proteins on the membranes were detected with an ECL detection kit (Thermo Scientific, WP20005, Waltham, MA, USA). The intensities of the protein bands were assessed using ImageJ 1.53 software (National Institutes of Health, NIH, USA).

### Statistical analysis

All experiments were conducted in triplicate GraphPad Prism 9 (GraphPad Software, Inc., La Jolla, CA, USA) and SPSS 26.0 software (International Business Machines Corporation, New York, NY, USA) were utilized for statistical and graphical analyses. Results are presented as mean ± SD. Comparisons between groups were performed using an unpaired two-tailed Student’s *t*-test. Spearman’s correlation analysis was conducted, using the correlation coefficient (R) to evaluate the strength of the relationship. Survival analysis was conducted using the Kaplan-Meier method. The Cox proportional hazards model was utilized to calculate the hazard ratios (HR) for each clinicopathological variable linked to overall survival (OS). In the OS analysis, factors such as non-breast cancer-related deaths and other confounding variables were taken into account. Chi-square tests were employed to examine the associations between NT5E expression levels and clinical features. *p < 0.05, **p < 0.01, or ***p < 0.001.

## Results

### Transcriptome analysis revealed that PD-L1 and NT5E have a correlation

**Figure 1:**
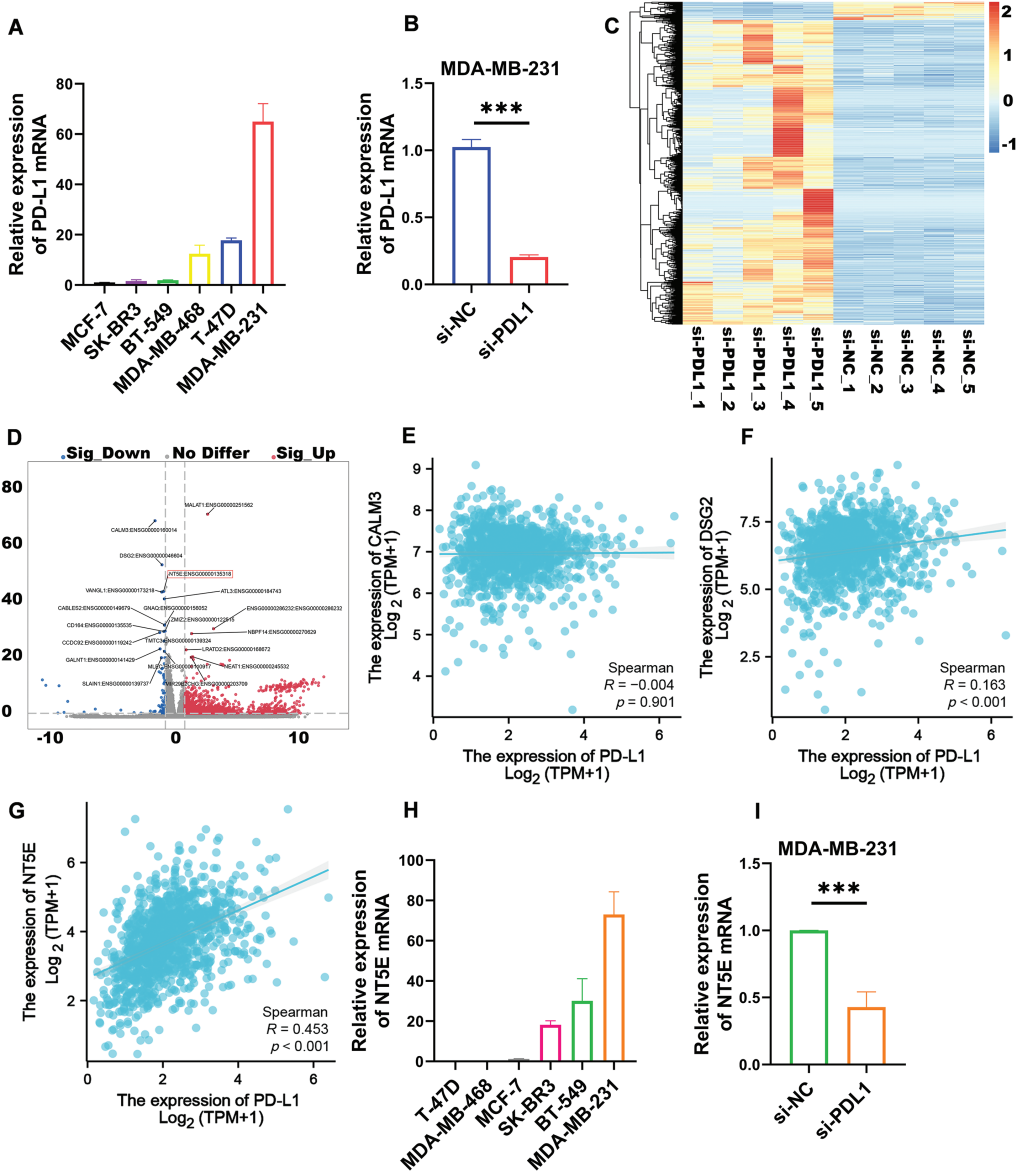
Transcriptomic analysis revealed that the expression of the differentially expressed gene NT5E was down regulated following PD-L1 gene knockout. (A) The TNBC cell line MDA-MB-231 demonstrates the highest levels of PD-L1 mRNA expression compared to other breast cancer cell lines (B) Silencing the PD-L1 gene using siRNA resulted in a decrease in PD-L1 mRNA expression in MDA-MB-231. (C) Differential genes obtained after PD-L1 gene silencing in MDA-MB-231. (D) Top 20 differential genes obtained after PD-L1 gene silencing in MDA-MB-231. (E) Correlation analysis of PD-L1 and CALM3 in breast cancer. (F) Correlation analysis of PD-L1 and DSG2 in breast cancer. (G) Correlation analysis of PD-L1 and NT5E in breast cancer. (H) MDA-MB-231 exhibits the highest expression of NT5E mRNA among breast cancer cell lines. (I) qRT-PCR showed that NT5E expression decreased after PD-L1 gene silencing. (*p* Significant Codes: ***p  <  0.001).

Utilizing real-time quantitative PCR, we evaluated PD-L1 expression in six common breast cancer cell lines: MDA-MB-231, T-47D, MDA-MB-468, BT-549, SK-BR-3, and MCF-7. The results revealed that MDA-MB-231 exhibited the highest PD-L1 mRNA expression (Fig. 1A), prompting its selection for subsequent experiments. We silenced the PD-L1 gene in MDA-MB-231 using si-PD-L1. Compared to si-NC control group, PD-L1 mRNA expression in si-PD-L1 group was significantly downregulated (0.20 ± 0.02 *vs*. 1.02 ± 0.06; *p* < 0.001; Fig. 1B). Subsequently, RNA-seq analysis we performed on PD-L1-silenced MDA-MB-231 to compare differentially expressed genes between si-PD-L1 and si-NC groups. The analysis identified 1477 differentially expressed genes comprising 83 downregulated and 1394 upregulated genes (Fig. 1C). A volcano plot was generated for the top 20 differentially expressed genes, with log_2_(FC) on the *x*-axis and −log_10_(*q*-value) on the *y*-axis, where blue and red sections represent significantly down-regulated and up-regulated genes, respectively (Fig. 1D). We focused on downregulated genes with relatively high expression levels, as they may be detectable in tumor tissues and could serve as potential therapeutic and diagnostic targets. Based on a comprehensive analysis, using the Spearman method, we analyzed the correlation in the TCGA database (Fig. 1E–G). The results indicated a significant positive correlation between PD-L1 and NT5E expression in breast cancer (R = 0.453, *p* < 0.001). We selected NT5E (log_2_FC = 1.18, *p* < 0.001), the third-ranked downregulated gene (the top two were excluded due to low R values), as the focus of our study, suggesting its potential importance in influencing breast cancer malignant progression. Quantitative real-time PCR was conducted to assess NT5E mRNA expression in the aforementioned breast cancer cell lines, with results indicating the highest expression in MDA-MB-231 cells (Fig. 1H). Further quantitative real-time PCR analysis after PD-L1 silencing in MDA-MB-231 cells demonstrated significant downregulation of NT5E mRNA expression (0.43 ± 0.11 *vs*. 1.00 ± 0.00; *p* < 0.001; Fig. 1I).

### NT5E and PD-L1 expression and prognostic analysis in breast cancer

**Figure 2:**
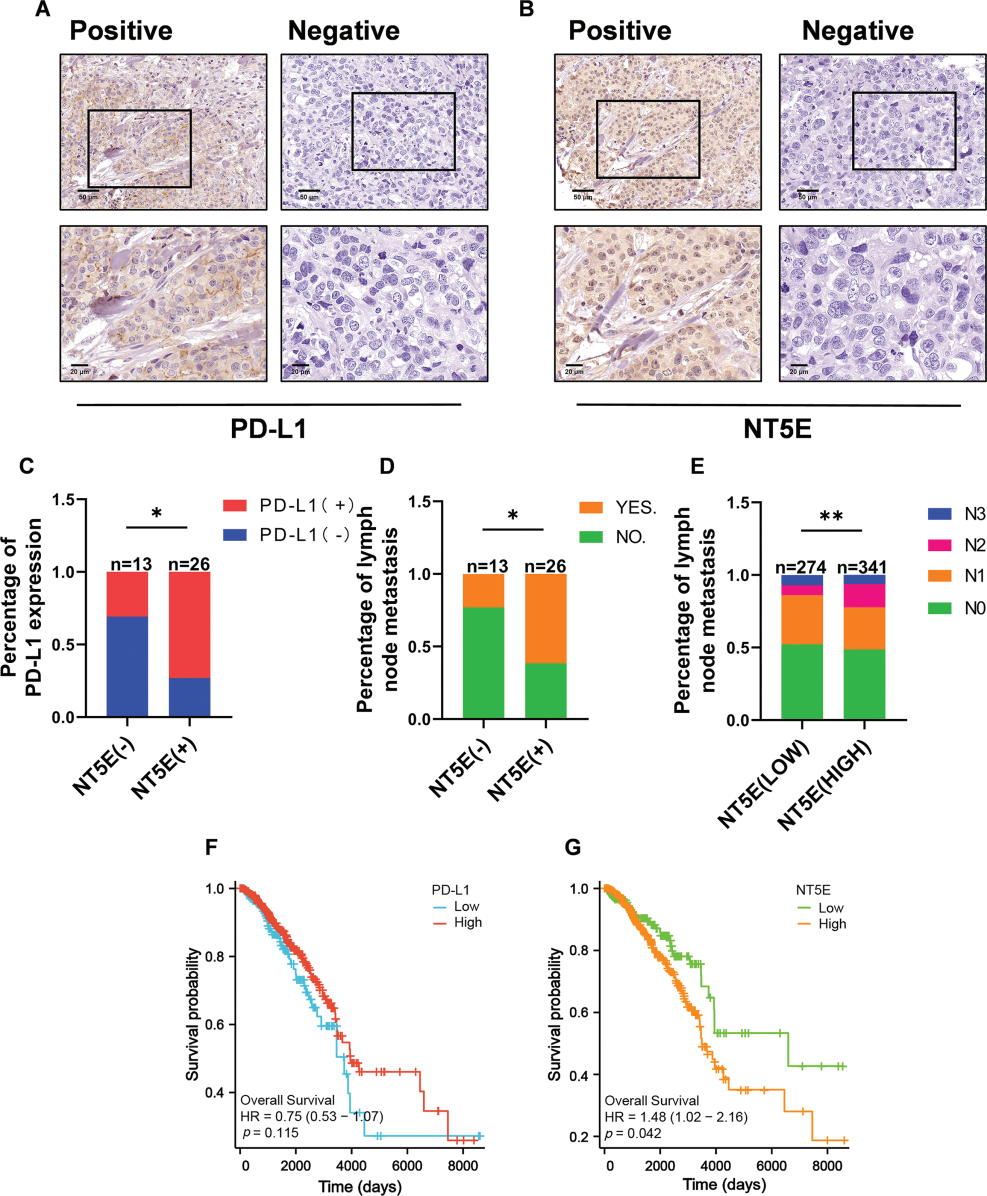
NT5E and PD-L1 expression and prognostic analysis in breast cancer tissues. (A) Representative immunohistochemical staining images of PD-L1 (magnification ×200, ×400). (B) Representative immunohistochemical staining images of NT5E in breast cancer tissues (magnification ×200, ×400). (C) The immunohistochemical indicate that NT5E protein expression is positively correlated with PD-L1 protein. (D) The immunohistochemical show that NT5E protein expression is associated with lymph node metastasis. (E) The database analysis results indicate that NT5E expression is associated with lymph node metastasis. (F) Kaplan-Meier survival curves were generated from the database to show that PD-L1 expression is not associated with prognosis. (G) Kaplan-Meier survival curves were generated from the database to show that NT5E expression correlates with prognosis (*p* Significant Codes: *p  <  0.05, **p  <  0.01).

To validate the expression of NT5E and PD-L1 proteins in breast cancer, immunohistochemical (IHC) staining was conducted on 39 breast cancer tissues from our clinical specimen bank to analyze the correlation between NT5E and PD-L1 (Fig. 2A,B). Results revealed that in our series, the positive and negative rates of NT5E expression in breast cancer tissues were 33.3% (13/39) and 66.7% (26/39), respectively. For PD-L1 expression, positive and negative rates were 41.03% (16/39) and 58.97% (23/39), respectively. A significant positive correlation was observed between NT5E and PD-L1 expression (χ^2^ = 6.412, *p* = 0.011) (Table 1 and Fig. 2C). Additionally, NT5E expression was significantly associated with lymph node metastasis in breast cancer patients. (χ^2^: 5.132, *p* = 0.023) (Table 2 and Fig. 2D). Analysis of RNA sequencing data from 615 breast cancer patients, obtained from the TCGA database, revealed that low and high NT5E expression accounted for 44.55% (274/615) and 55.45% (341/615), respectively. High NT5E expression was significantly associated with lymph node metastasis (χ^2^ = 12.361, *p* = 0.006) (Table 3) (Fig. 2E). Utilizing the Cox regression analysis, the Kaplan-Meier curve revealed that breast cancer patients exhibiting low NT5E expression experienced improved OS compared to those with elevated NT5E levels (HR = 1.48, 95% CI: 1.02–2.16, *p* = 0.042), suggesting NT5E’s potential as a prognostic indicator for breast cancer patients. No notable difference was found in the prognosis of breast cancer patients concerning PD-L1 expression (HR = 0.75, 95% CI: 0.53–1.07, *p* = 0.115, Fig. 2F,G).

**Table 1:** Correlation between NT5E and PD-L1 expression in breast cancer tissues (N = 39)

Patients	Expression of NT5E		*p* Value
	Low NT5E n = 13	High NT5E n = 26	
**PD-L1**			0.011
Negative	9	7	
Positive	4	19	

**Table 2:** The relationship between NT5E expression and breast cancer patient’s clinicopathological characteristics (N = 39)

Patients	Expression of NT5E		*p* Value
	Low NT5E n = 13	High NT5E n = 26	
**Age**			0.648
<=50	5	12	
>50	8	14	
**Tumor size**			0.819
≤2	6	11	
>2	7	15	
**TNM pathological stage**			0.477
I–II	10	16	
III	3	10	
**Lymph nodes involved**			0.023
Negative	10	10	
Positive	3	16	
**Intravascular tumor thrombus**			0.276
Negative	11	17	
Positive	2	9	
**ER**			1
Negative	5	10	
Positive	8	16	
**PR**			1
Negative	7	14	
Positive	6	12	
**HER2**			
Negative	7	18	0.345
Positive	6	8	
**Ki67**			0.361
≤30%	6	16	
>30%	7	10	
**Molecular subtype**			0.677
Luminal	5	12	
HER2	6	8	
TNBC	2	6	

Note: Estrogen receptors (ER), Progesterone receptors (PR), and Human epidermal growth factor receptor 2 (HER2).

**Table 3:** The relationship between NT5E expression and breast cancer patient’s clinicopathological characteristics (N = 615)

Patients	Expression of NT5E		*p* Value
	Low NT5E n = 274	High NT5E n = 341	
**Age**			0.589
<=60	158	204	
>60	116	137	
**Pathologic T stage**			0.540
T1	62	92	
T2	172	206	
T3	33	33	
T4	7	10	
**Pathologic N stage**			0.006
N0	143	166	
N1	93	99	
N2	19	55	
N3	19	21	
**Pathologic M stage**			1
M0	270	337	
M1	4	4	
**Pathologic stage**			0.207
Stage I	46	60	
Stage II	174	192	
Stage III	50	85	
Stage IV	4	4	
**ER status**			0.531
Negative	68	79	
Positive	205	262	
Indeterminate	1	0	
**PR status**			0.265
Negative	102	106	
Positive	171	233	
Indeterminate	1	2	
**HER2 status**			0.754
Negative	216	261	
Positive	56	78	
Indeterminate	2	2	
**Molecular subtype**			0.678
LumA	133	174	
LumB	55	61	
HER2	28	28	
Basal	51	64	
Indeterminate	7	14	

Note: Estrogen receptors (ER), Progesterone receptors (PR), and Human epidermal growth factor receptor 2 (HER2).

### Investigation of the biological functions of PD-L1 in breast cancer cell lines

**Figure 3:**
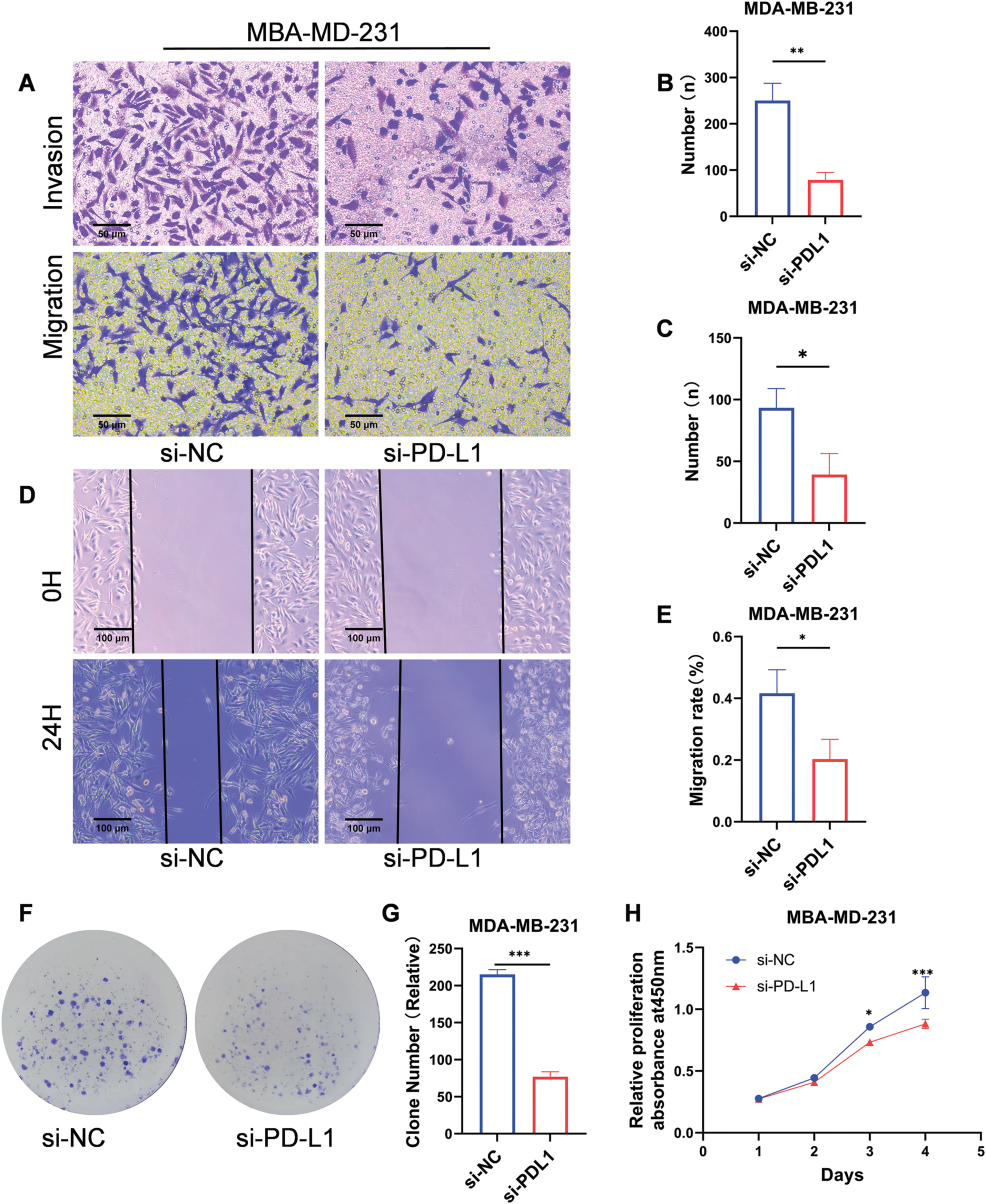
Biological functions of PD-L1 in breast cancer cells. (A–C) Reduced migratory (A, B) and invasive (A, C) capacities of cells transfected with si-PD-L1 were verified using Transwell migration and Matrigel invasion assays. (D, E) The wound healing assay validated decreased migration ability of cells after PD-L1 gene silencing. (F, G) The colony formation assay validated decreased proliferation ability of cells after PD-L1 gene silencing. (H) Reduced proliferation of cells after PD-L1 gene silencing was verified using CCK-8 assays (*p* Significant Codes: *p  <  0.05, **p  <  0.01, ***p  <  0.001).

MDA-MB-231 was selected for knockdown assay using a small RNA interference technique to further investigate the biological functions of PD-L1. Migration and invasion assays revealed that PD-L1 knockdown significantly reduced the migratory capacity (number of transmembrane cells: 78.67 ± 16.01 *vs*. 250 ± 37.36; *p* < 0.01; Fig. 3A,B) and decreased the invasive ability (number of transmembrane cells: 39.33 ± 17.01 *vs*. 93.33 ± 15.63; *p* < 0.05; Fig. 3A,C) of the cells compared to the si-NC group. Additionally, the wound healing assay further confirmed that PD-L1 knockdown diminished the migratory capacity of the cells (healing percentage: 20.34% ± 6.41% *vs*. 41.63% ± 7.63%; *p* < 0.05; Fig. 3D,E). Furthermore, the colony formation assay demonstrated that the size of colonies formed by PD-L1 knockdown cells was smaller than that of the control cells (77 ± 6.55 *vs*. 215 ± 6.55; *p* < 0.001; Fig. 3F,G). CCK-8 assay indicated that cell proliferation ability was significantly decreased on days 3 and 4 after PD-L1 knockdown compared with si-NC group (*p* < 0.001, Fig. 3H).

### In vitro validation of the relationship between NT5E and PD-L1

**Figure 4:**
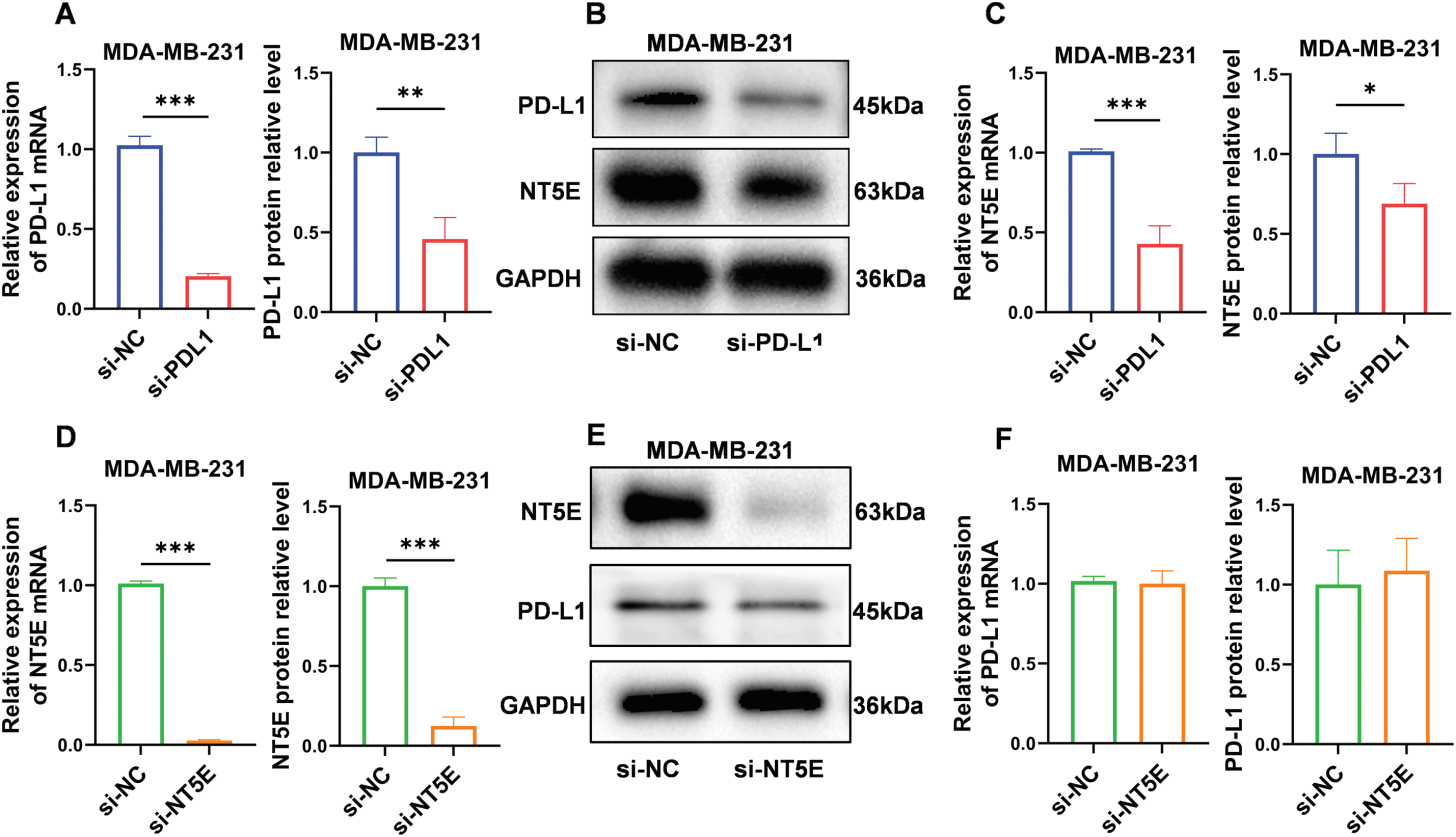
PD-L1 functions as an upstream regulator of NT5E expression. (A, B) Reduced expression of PD-L1 mRNA and protein after PD-L1 gene silencing by si-RNA method. (B, C) qRT-PCR and western blotting validated decrease in NT5E expression following PD-L1 gene knockout. (D, E) Reduced expression of NT5E mRNA and protein after NT5E gene silencing by si-RNA method. (E, F) qRT-PCR and western blotting verified that PD-L1 expression remained unchanged after NT5E knockdown (*p* Significant Codes: *p  <  0.05, **p  <  0.01, ***p  <  0.001).

To validate the relationship between PD-L1 and NT5E, we conducted gene knockdown experiments using small RNA interference technology on the MDA-MB-231 cell line. We initially confirmed successful knockdown of PD-L1 (mRNA level: as shown in Fig. 1B; protein level: 0.46 ± 0.14 *vs*. 1.00 ± 0.10; *p* < 0.01; Fig. 4A,B). Quantitative RT-PCR and western blotting analyses revealed that NT5E expression was significantly downregulated in si-PD-L1 cells compared to si-NC control group (mRNA level: as shown in Fig. 1I; protein level: 0.69 ± 0.13 *vs*. 1.00 ± 0.13; *p* < 0.05; Fig. 4B,C) Subsequently, we performed gene knockdown of NT5E in MDA-MB-231 using small RNA interference technology. We verified that NT5E was significantly downregulated following NT5E silencing (mRNA level: 0.03 ± 0.01 *vs*. 1.00 ± 0.00; *p* < 0.001; protein level: 0.12 ± 0.06 *vs*. 1.00 ± 0.05; *p* < 0.001; Fig. 4D,E). Quantitative RT-PCR and Western blotting analyses indicated no significant change in PD-L1 expression in si-NT5E cells compared to the si-NC control group (mRNA: 1.00 ± 0.00 *vs*. 1.00 ± 0.08; *p* = 0.99; protein level: 1.09 ± 0.20 *vs*. 1.00 ± 0.22; *p* = 0.63; Fig. 4E,F). The above results indicate that PD-L1 is an upstream gene influencing NT5E expression.

### PD-L1 influences NT5E expression via the mitogen-activated protein kinase (MAPK)/ERK pathway

**Figure 5:**
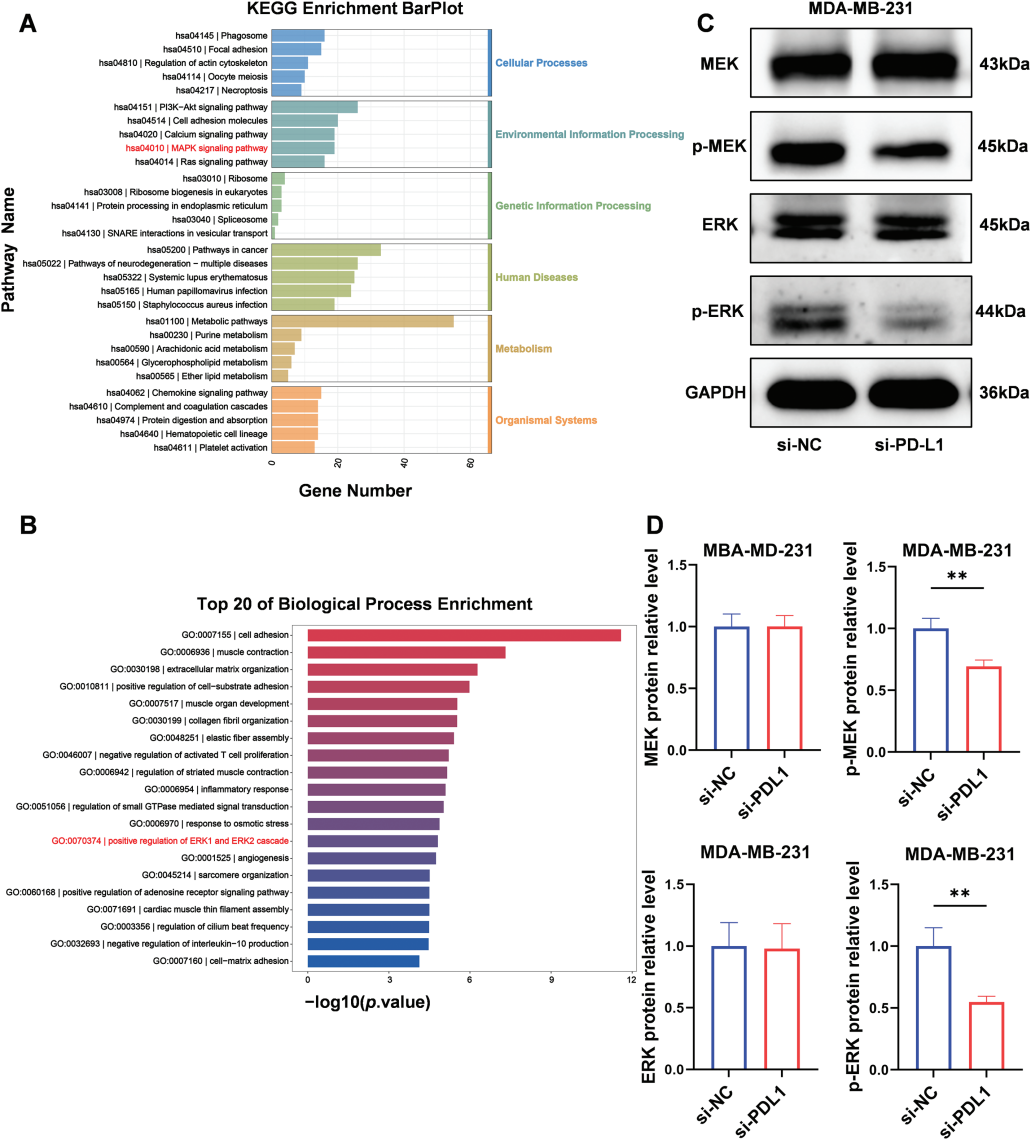
PD-L1 influences NT5E expression through the MAPK/ERK pathway. (A, B) Enrichment analysis for representative GO_BP and KEGG pathways after transfected with PD-L1 siRNA. (C, D) After transfected with PD-L1 siRNA, p-MEK and p-ERK proteins expression was downregulated, while MEK and ERK remained unchanged (*p* Significant Codes: **p  <  0.01).

We performed pathway enrichment analysis utilizing the Kyoto Encyclopedia of Genes and Genomes (KEGG) and GO analysis on aforementioned RNA-seq data. The KEGG pathway enrichment analysis indicated that this process is regulated by the MAPK signaling pathway (Fig. 5A). The GO analysis revealed that the protein expression of phosphorylated extracellular signal-regulated kinases (ERK1 and ERK2) was affected in the PD-L1 knockdown cell lines (Fig. 5B). To validate these findings, we conducted a Western blotting analysis. Compared to the si-NC control group, the expression of p-MEK and p-ERK proteins was significantly downregulated in si-PD-L1 cells (p-MEK: 0.69 ± 0.05 *vs*. 1.00 ± 0.08; *p* < 0.01; p-ERK: 0.55 ± 0.05 *vs*. 1.00 ± 0.15; *p* < 0.01; Fig. 5C,D). In contrast, the total levels of MEK and ERK proteins exhibited no significant changes (MEK: 1.00 ± 0.10 *vs*. 1.00 ± 0.09; *p* = 0.98; ERK: 0.98 ± 0.20 *vs*. 1.00 ± 0.19; *p* = 0.91; Fig. 5C,D).

### Investigation of the biological functions of NT5E in breast cancer cell line

**Figure 6:**
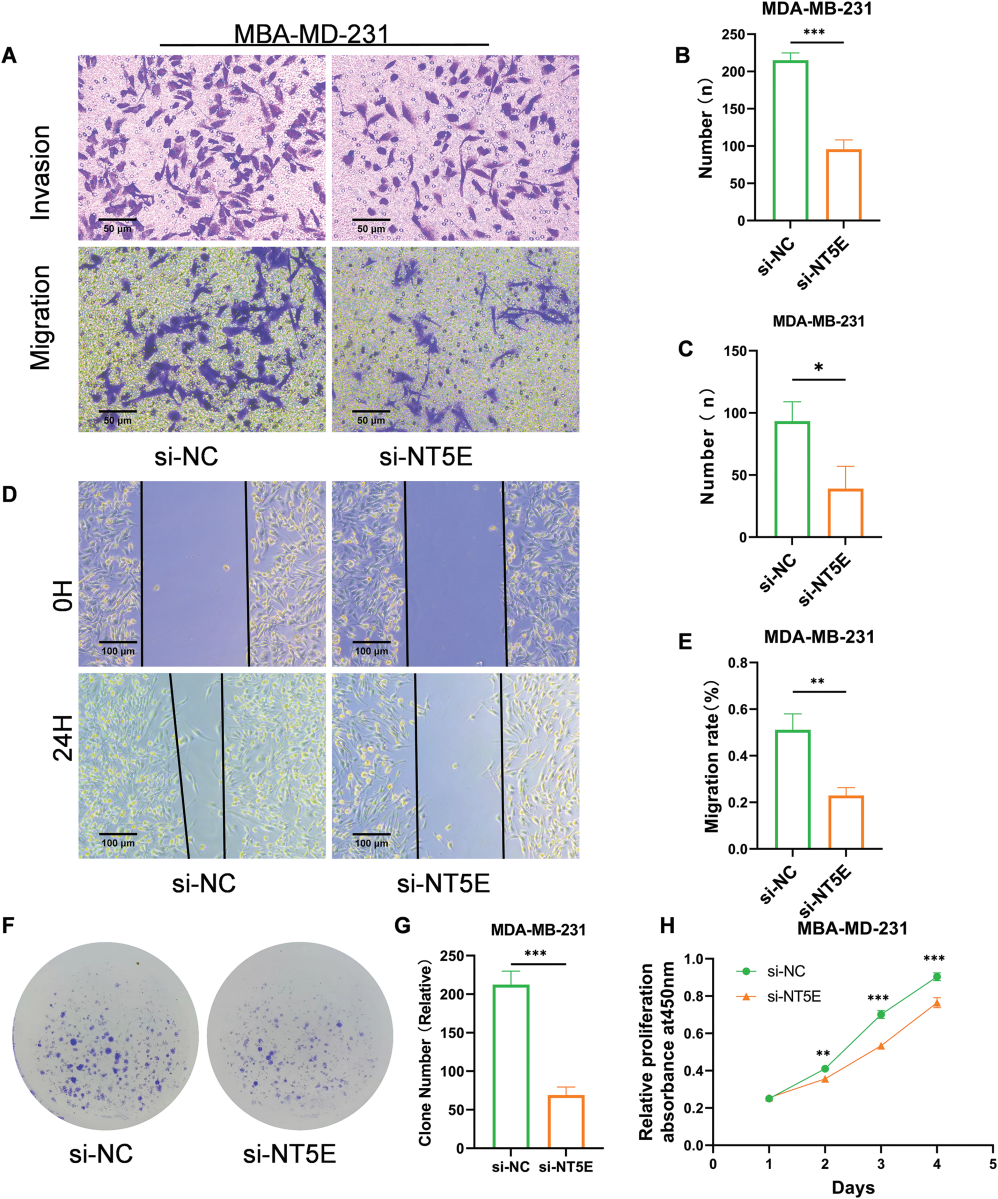
Biological functions of NT5E in breast cancer cells. (A–C) Reduced migratory (A, B) and invasive (A, C) capacities of cells transfected with si-NT5E were verified using Transwell migration and Matrigel invasion assays. (D, E) The wound healing assay validated decreased migration ability of cells after NT5E gene silencing. (F, G) The colony formation assay validated decreased proliferation ability of cells after NT5E gene silencing. (H) Reduced proliferation of cells after NT5E gene silencing was verified using CCK-8 assays (*p* Significant Codes: *p  <  0.05, **p < 0.01, ***p  < 0.001).

To further investigate the biological functions of NT5E, MDA-MB-231 was selected for knockdown assay using a small RNA interference technique. NT5E knockdown significantly reduced the migratory capacity of the cells (number of transmembrane cells: 95.67 ± 12.50 *vs*. 215 ± 9.85; *p* < 0.001; Fig. 6A,B) and their invasive ability (number of transmembrane cells: 39 ± 18 *vs*. 93.3 ± 15.63; *p* < 0.05; Fig. 6A,C). Moreover, NT5E knockdown diminished the cell migratory capacity (healing percentage: 22.95% ± 3.34% *vs*. 51.11% ± 6.83%; *p* < 0.01; Fig. 6D,E). In addition, the spheres formed by NT5E knockout cells were smaller compared to those formed by the control cells (69 ± 10.54 *vs*. 212.33 ± 17.62; *p* < 0.001; Fig. 6F,G). The results of the CCK-8 assay demonstrated a significant reduction proliferation ability on days 2, 3, and 4 following the knockdown of NT5E (*p* < 0.001, Fig. 6H).

## Discussion

The function of PD-L1 in facilitating tumor cell evasion from immune surveillance has been thoroughly examined [18,19]. Furthermore, elevated PD-L1 expression correlates with poor patient prognosis [20,21]. An increasing number of clinical studies have shown that treatments aimed at PD-1/PD-L1 significantly enhance overall survival in patients with advanced cancer [22,23]. These treatments have also shown clinical effectiveness in breast cancer, especially among patients with TNBC. However, some patients experience delayed responses, excessive progression, or develop resistance, resulting in a substantial proportion of patients not benefiting from these treatments [24–26]. Emerging evidence has illuminated the tumor cell-intrinsic functions of PD-L1, revealing its autonomous involvement in regulating multiple oncogenic processes including cellular proliferation, metastatic progression, therapeutic resistance, and metabolic reprogramming, through mechanisms distinct from its well-characterized immune checkpoint functions.

This research presents the initial evidence that PD-L1 regulates NT5E expression through the MAPK/ERK signaling pathway, thereby promoting breast cancer cell proliferation and metastatic. Initially, we selected the MDA-MB-231 (TNBC), which exhibits the highest PD-L1 expression among six common breast cancer cell lines, for subsequent experiments. RNA-seq analysis was performed on the MDA-MB-231, after PD-L1 silencing. By comparison, we identified NT5E as the third-ranked significantly differentially expressed gene for further study. Subsequently, we validated the mRNA levels in the PD-L1-silenced MDA-MB-231 cell line with consistent results. Additionally, analysis of online databases for breast cancer indicated a correlation between PD-L1 expression and NT5E A previous study conducted [27], specific antibody staining for CD39, CD73 (NT5E), and PD-L1 on MDA-MB-231, observing multiple double-positive populations, with the highest proportion of cells co-expressing NT5E and PD-L1. However, that study did not conduct an in-depth investigation of this aspect. Therefore, to explore the potential underlying mechanism, we selected NT5E as the focus of further research.

NT5E is a glycosylphosphatidylinositol-anchored enzyme found on the surface of cells in normal tissues. In the tumor microenvironment, NT5E demonstrates both enzymatic and non-enzymatic functions. The enzymatic activity of NT5E converts AMP to adenosine (ADO), which is important in a range of physiological and pathophysiological processes. Extracellular ADO can influence the tumor immune microenvironment through multiple pathways and is crucial in tumor immune tolerance. In addition to its enzymatic activities, NT5E modulates pathways, including the EGFR/Akt and VEGF/Akt pathways, which negatively affect anti-tumor immunity. This regulation is crucial for tumor cell proliferation, angiogenesis, and apoptosis [28,29]. Additionally, NT5E can reduce intercellular adhesion by regulating cadherin-1 and vimentin, thereby inducing epithelial-mesenchymal transition (EMT) and conferring an “invasive phenotype” to tumor cells. Consequently, NT5E is instrumental in facilitating tumor metastasis. The upregulation of NT5E expression is predominantly mediated by hypoxia-inducible factors within the tumor microenvironment, coupled with a complex network of pro-inflammatory cytokines and soluble mediators that collectively establish a permissive niche for its transcriptional activation. Accumulating evidence from contemporary oncological research has identified NT5E as a frequently upregulated ectoenzyme across diverse epithelial malignancies, with particularly pronounced overexpression observed in mammary carcinoma, colorectal adenocarcinoma, prostatic neoplasms, and ovarian epithelial cancers, suggesting its potential as a pan-cancer biomarker [30].

We subsequently validated NT5E expression in 39 breast cancer samples from our hospital, revealing a significant positive correlation between NT5E and PD-L1 expression in pathological sections. A seminal investigation by [31] employed comprehensive immunohistochemical profiling of 53 clinical specimens, establishing a statistically robust association between NT5E and PD-L1 co-expression patterns in tumor tissues, thereby providing compelling clinical evidence supporting their potential functional interplay in cancer biology. Notably, our immunohistochemical findings showed a correlation between elevated NT5E expression and the presence of lymph node metastasis. Analysis of 615 breast cancer samples from our database further confirmed this association. Additionally, analysis of the database indicated that patients exhibiting elevated NT5E expression experienced a worse prognosis. Loi et al. examined gene expression data from more than 6000 patients with TNBC and found that elevated NT5E expression correlates with a worse prognosis [32]. These clinical characteristics imply a possible oncogenic role for NT5E in breast cancer, especially in TNBC. However, the database analysis revealed no correlation between PD-L1 expression and patient prognosis. Considering potential confounding factors in database information that could influence PD-L1 expression beyond tumor cells, we selected MDA-MB-231 for functional validation. The results indicated that PD-L1 facilitated the proliferation and metastasis of human breast cancer cells, aligning with observations that PD-L1 enhances the proliferation of non-small cell lung cancer (NSCLC) cells [8]. To explore the precise relationship and underlying mechanisms between NT5E and PD-L1, we conducted dual validation at both gene and protein levels. Silencing PD-L1 significantly downregulated NT5E expression, while silencing NT5E did not notably change PD-L1 expression, indicating PD-L1 as an upstream gene influencing NT5E expression. To elucidate underlying mechanisms, we performed KEGG and GO analyses on RNA-seq data from MDA-MB-231 after PD-L1 gene silencing. Enrichment analysis highlighted the MAPK/ERK pathway, which we subsequently validated at the protein level.

The MAPK signaling pathway is essential for regulating proliferation, differentiation, apoptosis, and stress response. It is linked with the progression of multiple tumors, including breast cancer [33,34]. Our study revealed that pMEK and pERK protein expressions were downregulated following PD-L1 knockdown, confirming that the ERK/MAPK pathway is regulated by PD-L1. Additionally, we utilized the NT5E-silenced expressing TNBC cell line MDA-MB-231 to further validate its malignant function *in vitro*. This observation regarding NT5E aligns with recent findings in breast cancer research [27].

This study demonstrates that PD-L1 partially regulates NT5E expression through the MAPK/ERK pathway, contributing to breast cancer progression. Research has demonstrated that NT5E contributes to anthracycline resistance and is associated with unfavorable prognosis in TNBC. Moreover, inhibiting NT5E markedly augmented doxorubicin-induced anti-tumor immune responses and prolonged survival in a metastatic breast cancer model [32]. The NT5E inhibitor, α, ß-methylene adenosine-5′-diphosphate (APCP), significantly suppressed TNBC cell growth both *in vitro* and *in vivo* [35]. A bifunctional PD-L1/CD73(NT5E) small-molecule inhibitor was developed, exhibiting high PD-L1 and NT5E inhibitory activity in a melanoma model. These studies collectively suggest the potential of targeting NT5E in cancer immunotherapy [15]. Consequently, NT5E is regarded as a tumor-associated biomarker with potential therapeutic value for future breast cancer treatments. However, additional research is required. Although this study advances our comprehension of the interplay between NT5E and PD-L1 in breast cancer, it also presents limitations that should be taken into account.

This study presents several limitations. Firstly, the database results provide only partial information regarding NT5E at the gene level and its correlation with PD-L1, potentially not fully representing the breast cancer landscape in China. While single-cell RNA sequencing technology and clinical pathological slice immunohistochemical analyses may address this issue, a larger sample size is necessary for further validation. Secondly, the key signaling pathways involved in this process require more comprehensive experimental studies to elucidate their molecular mechanisms. Thirdly, the *in vitro* findings on cellular proliferation, invasion, and migration functions necessitate validation through *in vivo* animal model experiments.

In conclusion, this study presents compelling evidence that elevated NT5E expression correlates with reduced OS in breast cancer patients. The research proposes a potential mechanism whereby PD-L1 modulates NT5E expression through the MAPK/ERK signaling pathway, contributing to breast cancer progression. These results enhance our understanding of the underlying biological mechanisms and may offer new avenues for prognostic assessment and therapeutic interventions. The results could potentially guide future clinical strategies for breast cancer diagnosis and treatment, opening novel directions for research and clinical applications.

## Data Availability

The raw datasets and analytical materials are archived and maintained by the corresponding authors, and will be made accessible to qualified researchers upon submission of a formal data access request that outlines the intended scientific use.

## Appendix A

**Table A1:** Clinicopathological characteristics of the patient (N = 39)

Characteristics	Patients	%
**Age (years), median (range)**	52 (38–81)	
<=50	17	43.590
>50	22	56.410
**Tumor size**		
≤2	17	43.590
>2	22	56.410
**TNM patholohical stage**		
I–II	26	66.667
III	13	33.333
**Lymph nodes involved**		
Negative	20	51.282
Positive	19	48.718
**Intravascular tumor thrombus**		
Negative	28	71.795
Positive	11	28.205
**ER**		
Negative	15	38.462
Positive	24	61.538
**PR**		
Negative	21	53.846
Positive	18	46.154
**HER2**		
Negative	25	46.153
Positive	14	35.897
**KI67**		
≤30%	22	56.410
>30%	17	43.590
**Molecular subtype**		
Luminal	17	43.590
HER2	14	35.897
TNBC	8	20.513

**Table A2:** Clinicopathological characteristics of the patient (N = 615)

Characteristics	Patients	%
**Age (years)**		
<=60	362	58.86
>60	253	41.14
**Pathologic T stage**		
T1	154	25.04
T2	378	61.46
T3	66	10.73
T4	17	2.764
**Pathologic N stage**		
N0	309	50.24
N1	192	31.22
N2	74	12.03
N3	40	6.504
**Pathologic M stage**		
M0	607	98.70
M1	8	1.301
**Pathologic stage**		
Stage I	106	17.24
Stage II	366	59.51
Stage III	135	21.95
Stage IV	8	1.301
**ER status**		
Negative	147	23.90
Positive	467	75.93
Indeterminate	1	0.163
**PR status**		
Negative	208	33.82
Positive	404	65.69
Indeterminate	3	0.488
**HER2 status**		
Negative	477	77.56
Positive	134	21.78
Indeterminate	4	0.650
**Molecular subtype**		
LumA	307	49.918
LumB	116	18.86
HER2	56	9.106
Basal	115	18.70
Indeterminate	21	3.414
